# The balance between photosynthesis and respiration explains the niche differentiation between *Crocosphaera* and *Cyanothece*

**DOI:** 10.1016/j.csbj.2022.11.029

**Published:** 2022-11-17

**Authors:** Takako Masuda, Keisuke Inomura, Meng Gao, Gabrielle Armin, Eva Kotabová, Gábor Bernát, Evelyn Lawrenz-Kendrick, Martin Lukeš, Martina Bečková, Gábor Steinbach, Josef Komenda, Ondřej Prášil

**Affiliations:** a Institute of Microbiology, The Czech Academy of Sciences, Opatovický mlýn, 37901 Třeboň, Czech Republic; b Graduate School of Oceanography, University of Rhode Island, Narragansett, RI 02882, USA; c Balaton Limnological Research Institute, Eötvös Loránd Research Network (ELKH), Tihany, Hungary; d Cellular Imaging Laboratory, Biological Research Center, Eötvös Loránd Research Network (ELKH), Szeged, Hungary

**Keywords:** UCYN-B, UCYN-C, Niche separation, Carbon consumption

## Abstract

*Crocosphaera* and *Cyanothece* are both unicellular, nitrogen-fixing cyanobacteria that prefer different environments. Whereas *Crocosphaera* mainly lives in nutrient-deplete, open oceans, *Cyanothece* is more common in coastal, nutrient-rich regions. Despite their physiological similarities, the factors separating their niches remain elusive. Here we performed physiological experiments on clone cultures and expand upon a simple ecological model to show that their different niches can be sufficiently explained by the observed differences in their photosynthetic capacities and rates of carbon (C) consumption. Our experiments revealed that *Cyanothece* has overall higher photosynthesis and respiration rates than *Crocosphaera*. A simple growth model of these microorganisms suggests that C storage and consumption are previously under-appreciated factors when evaluating the occupation of niches by different marine nitrogen fixers.

## Graphical abstract

**Figure:**
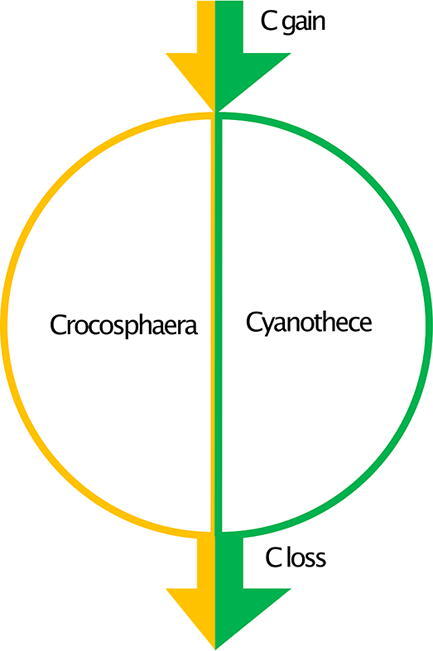

## 1 Introduction

Nitrogen (N) is the major limiting factor for primary productivity in the subtropical and tropical ocean gyres. In such regions, specialized prokaryotes, 'dinitrogen (N_2_) fixers' or 'diazotrophs', are able to use N in the most abundant form on Earth and in seawater, N_2_ gas. Diazotrophs utilize the nitrogenase enzyme which cleaves the strong triple bond of the N_2_ molecule to form bioavailable ammonium (NH_4_^+^). Thus, N_2_ fixation introduces a source of new bioavailable N to surface waters and is considered the most important external source of N to the ocean, supporting ocean productivity and biological pump [2], [15], [16], [39]. Marine autotrophic unicellular diazotrophs play a key role in biogeochemical cycles [44].

Marine autotrophic unicellular diazotrophs are phylogenetically divided into two groups. The unicellular group B (UCYN-B) is most closely related to *Crocosphaera watsonii* [24]*.* UCYN-B lives singly, colonially or in symbioses with a large chain-forming diatom (*Climacodium frauenfeldianum*) [4], [5] containing cultivated strains (i.e. *C. watsonii* WH8501, WH0003, PS0609) [25], [42]. The unicellular group C (UCYN-C) is the group identified by *nif*H sequence and is most closely related to the free-living unicellular diazotroph *Cyanothece* sp. strain ATCC51142 [35]. UCYN-B widely distributes in oligotrophic sub-tropical and tropical open ocean [27], [40], while, the distribution of UCYN-C is reported in coastal regions [37], [41].

The distribution of these diazotrophs is constrained by the growth capacity, which is supported by capacities of photosynthesis and N_2_ fixation. Since nitrogenase is inactivated by the oxygen [12], [14], both of these groups segregate these processes by temporal separation: restrain N_2_ fixation to the night when oxygen evolution of light-dependent photosynthesis is absent [1], [45]. The timing of these processes is primarily regulated by the circadian clock [6], and the nocturnal nitrogen fixation is fueled by carbon (C) accumulated during the light period [8], [36]. Given the above, how is niche separation between *Crocosphaera* and *Cyanothece* related to their relationship between photosynthesis and N_2_ fixation?

In this study, we performed physiological experiments to investigate the capacities of photosynthesis and N_2_ fixation in *Crocosphaera watsonii* WH8501 and *Cyanothece* sp. ATCC51142 under optimum growth conditions. Further, we elaborated on a simple ecological model to show that their different niches can be sufficiently explained by the observed differences in their photosynthetic capacities and rates of C consumption.

## 2 Materials and methods

We obtained *Crocosphaera watsonii* WH8501 (hereafter referred to as *Crocosphaera*) from the culture collection of the Royal Netherlands Institute of Sea Research in Yerseke (Strain CCY 0601) and maintained it in YBC-II medium without any enriched combined nitrogen source [7] at 28 °C in flat panel photobioreactors (FMT150; Photon System Instruments, Drásov, Czech Republic) [29]. Light intensity followed a sinusoidal 12:12 h light:dark cycle with a maximum irradiance of 400 µmol photons m^−2^ s^−1^ in the middle of the light period. *Cyanothece* sp. American Type Culture Collection (ATCC) 51142 (hereafter referred to as *Cyanothece*) has been recently renamed to *Crocosphaera subtropica* [23]. We maintained it under similar conditions as *Crocosphaera*, but grew it in ASP-2 media without NaNO_3_ [32] and set the maximum irradiance to 130 µmol photons m^−2^ s^−1^. The chosen light intensities represent the optimum light conditions of these two species based on the corresponding light saturation intensities for carbon incorporation, Ek_C_, which were 331 µmol quanta m^−2^ s^−1^ and 88 µmol quanta m^−2^ s^−1^ for *Crocosphaera* and *Cyanothece*, respectively (Table 1).

**Table 1:** Light or Dark phase accumulated chlorophyll normalized rates of ETR, O_2_ evolution, respiration, maximum carbon incorporation (P_m_^B^), N_2_ fixation and the efficiency of carbon fixation at light saturated (*Φ_max_*), and light limited (*Φ_lim_*), percentage of electron devoted for N_2_ fixation compared to electron transport during the day.

Parameters	Units	*Crocosphaera*		*Cyanothece*	
		Day	Night	Day	Night
E_K_C_	µmol quanta m^−2^ s^−1^	331	ND	88	ND
ETR_max_	µmol e (µg Chl)^−1^ 12 h^−1^	7.58	ND	13.62	ND
O_2_ evolution	µmol O_2_ (µg Chl)^−1^ 12 h^−1^	2.04	1.14	3.09	2.22
Respiration	µmol O_2_ (µg Chl)^−1^ 12 h^−1^	0.51	1.19	1.10	0.92
P_m_^B^	µmol C (µg Chl)^−1^ 12 h^−1^	1.66	ND	1.01	ND
N_2_ fixation	µmol N_2_ (µg Chl)^−1^ 12 h^−1^	0.00	0.08	0.00	0.09
Necessary e for N_2_ fixation	µmol e (µg Chl)^−1^ 12 h^−1^		0.62		0.75
ETR at given irradiance	µmol e (µg Chl)^−1^ 12 h^−1^	6.80		8.03	
% of electrons devoted to N_2_ fixation	%		9.1		9.3

We used the optical densities of the cultures at 735 nm (OD_735_) as an indicator of cell density. The photobioreactor monitored and averaged the OD_735_ data over every 1 or 5 min intervals and we normalized them to the OD_735_ values recorded at 1 h after the onset of the light phase (1L). Based on our earlier studies [20], [26], [34], we considered the increase in OD_735_ during the light phase as a proxy for C accumulation by photosynthesis. In contrast, the decrease in OD_735_ at the end of the light phase and also during the dark phase as a proxy for C consumption by respiration (see Discussion).

To determine the rates of N_2_ fixation by an acetylene reduction assay ([46]), we dispensed 5 mL of cell suspensions into HCl-rinsed glass vials (n = 3). After sealing with a septum, we injected 10 mL of acetylene gas (99.7 % [v/v]; Linde Gas) into each vial by replacing the same volumes of the headspace. The samples were incubated at 28 °C in the dark for 1 h. We took subsamples from the headspace immediately after acetylene addition and also at the end of the incubation period to determine their ethylene content with a flame ionization gas chromatograph (HRGC 5300, Carlo Erba Instruments, Strumentazione, Italy). We calculated the rate of ethylene production according to Breitbarth [3] and converted it to rate of N_2_ fixation using a theoretical molar ratio of acetylene reduction to cellular N_2_ reduction of 4:1 [28].

Using a Clark-type electrode combined with a DW2/2 electrode chamber (Hansatech, UK), we determined O_2_ evolution and respiration rates at 28 °C in the presence of 5 mM sodium bicarbonate. Depending on the culture cell density, we spun down different culture volumes by 10 min of centrifugation at 7500 g and re-suspended the pellet in 2.7 mL fresh medium. We calculated the rate of gross O_2_ evolution as a difference of net O_2_ evolution measured at a saturating irradiance of 600 µmol photons m^−2^ s^−1^ (KL1500, Schott, Mainz, Germany) and the of respiratory O_2_ consumption measured in the dark right after the light exposure.

To determine electron transport rates, we took aliquots (2 mL) from the photobioreactor after 1, 3, 5, 7, 9 and 11 h into the light period (hereafter referred to as (1L, 3L, 5L 7L, 9L and 11L) and transferred them into the measuring FastAct head of the benchtop FastOcean fast repetition rate FRR fluorometer (Chelsea Technologies Group, West Molesey, UK). We obtained photosynthesis-irradiance (P-E) curves by exposing the cells to increasing irradiances from 0 to 1495 µmol photons m^−2^ s^−1^ with 11 steps. Absolute electron transport rates (ETR) normalized to Chl *a* concentration (µmol electrons (µg Chl *a*)^−1^ h^−1^) were calculated according to the “absorption” method of [30] as:

(1) $${\mathrm{ETR}}_{\mathrm{Chl}}=\frac{F_{m}∙F_{o}}{\left(F_{m}-F_{o}\right)}∙\frac{\left(Fm^{\prime }-F^{\prime }\right)}{F_{m^{\prime }}}∙\frac{E∙K_{A}}{\left[Chla\right]}∙\frac{1}{3600}$$

where F_o_, and F_m_, are the minimum and maximum Chl *a* fluorescence in the dark, F′ and F_m_′ are the steady-state and maximal Chl *a* fluorescence measured at given light intensity, E is the intensity of the irradiance (in µmol photons m^−2^ s^−1^), K_A_ is the instrument-specific calibration factor (11800 m^−1^), 3600 is factor to convert seconds to hours, and [Chl *a*] is the Chl *a* concentration (in mg/m^3^). Then, we estimated the maximum electron transport rates (ETR_max_) by fitting the data to the model of [11].

To determine carbon incorporation of ^14^C-carbon, we collected 1 mL subsamples which were inoculated with ^14^C-labelled sodium bicarbonate (MP Biochemicals, CA, USA) at a concentration of 0.5 µCi/mL [17], and placed into a photosynthetron at 28 °C for 30 min at irradiances of 0 to 1528 μmol photons^−2^ s^−1^. Carbon uptake was then terminated by addition of 50 µL formaldehyde. Subsequently, we acidified samples with 250 µL of 3 N HCl and placed them onto an orbital shaker overnight to purge off unincorporated label. For counting radioactive decay, we added 5 mL of Ecolite liquid scintillation cocktail (MP Biochemicals, CA, USA) to each sample and placed them in a scintillation counter (PerkinElmer, MA, USA) for counting. The resultant rates of carbon incorporation were normalized to Chl *a* concentration to obtain the assimilation number, P_m_^B^, (in μmol C (μg Chl *a*)^−1^h^−1^).

We then calculated the electron demand for carbon assimilation *Φ*, either as the ratio of ETR_max_ and P_m_^B^ under saturating irradiance as *Φ_max_* = ETR_max_ / P_m_^B^ [22], or as the ratio of the initial slopes of ETR and carbon incorporation under non-saturating light irradiances, *Φ_Lim_ = α^ETR^ / α^C^,* where *α^ETR^* is the Chl *a* normalized absorption coefficient for electron transport rate (*α^ETR^,* µmol e (µg Chl *a*)^−1^ h^−1^ (µmol quanta m^−2^ s^−1^)^−1^) and *α^C^* is the Chl *a* normalized absorption coefficient for carbon incorporation (*α^C^*, µmol C (µg Chl *a*)^−1^ h^−1^ (µmol quanta m^−2^ s^−1^)^−1^).

For analysis of membrane proteins and their complexes, we isolated cyanobacterial membranes by breaking the cells with glass beads followed by differential centrifugation [21]. Afterwards, we solubilized the membranes in 1 % *n*-dodecyl maltoside (DM) and solubilized proteins and we separated their complexes by clear native polyacrylamide gel electrophoresis (CN PAGE). For assessment of the standard D1 (sD1) and rogue D1 (rD1) protein content, the membranes were analyzed in denaturing 12–20 % linear gradient polyacrylamide gel containing 7 M urea. We stained the gel with SYPRO Orange and transferred the separated proteins onto a polyvinylidene difluoride (PVDF) membrane. We incubated this membrane with specific primary antibodies against sD1 or rD1, then with a secondary antibody-horseradish peroxidase conjugate (Sigma, St. Louis, MO, USA) and specific signal of each protein was developed in the presence of chemiluminiscent substrate Immobilon Crescendo (Merck, USA).

### 2.1 Growth model

We modelled the growth rates of the studied microorganisms according to Eq. (2) (CFM-CC: Cell Flux Model of *Crocosphaera* and *Cyanothece*). It expresses growth rate $\mu_{i\;}$:

(2) $$\mu_{i\;}=P_{\mathrm{Max}}^{i}f_{N}-m_{i}$$

(unit: d^−1^, where *i* indicates microorganism, i.e., either *Crocosphaera* (*Cro*) or *Cyanothece* (*Cya*)). For microorganism *i*, we assumed that growth rate increases with both the maximum photosynthesis rate ($P_{\mathrm{Max}}^{i}$, unit: d^−1^) and the nutrient repletion factor ($f_{N}$, dimensionless unit) in the medium. The term $P_{\mathrm{Max}}^{i}f_{N}$ thus represents the rate of photosynthesis, which agrees with the general observation that nutrient repletion positively affects the rate of photosynthesis. The $f_{N}$ value represents the level of environmental nutrient repletion taking values from 0 (deplete environment) to 1 (replete environment). The equation also consists of a constant respiration rate ($m_{i}$, unit: d^−1^), which decreases the overall growth rate. Based on our experimental results (Fig. 2), we assigned higher photosynthesis $P_{\mathrm{Max}}^{i}$ and respiration $m_{i}$ rates to *Cyanothece* than to *Crocosphaera*. The list of parameters and parameter values are given in Tables S1 and S2, respectively.

## 3 Results and discussion

**Fig. 1:**
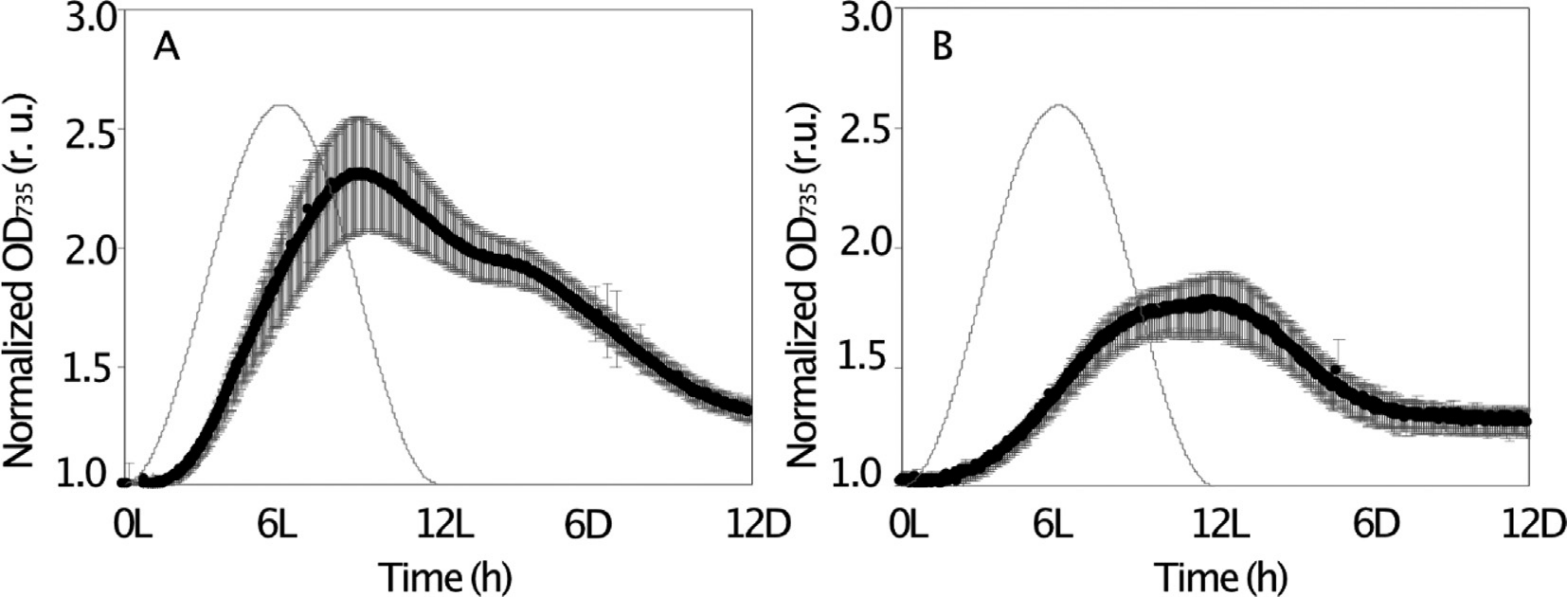
Diel change of the optical density at 735 nm (OD_735_) in *Crocosphaera* (A) and *Cyanothece* (B), where OD_735_ was normalized to OD_735_ values of 1 h into the light period (denoted) 1L. Closed circle shows the average with standard deviation (n = 6). The grey line represents the diagram of relative light intensity in relative units (r. u.).

In *Crocosphaera*, the normalized OD_735_ values increased up to ∼2.3 fold of the initial value at 9L, declining thereafter continuously until the end of the dark period to a final value of ∼1.4 fold of the initial value (Fig. 1A). In *Cyanothece* on the other hand, the corresponding normalized OD_735_ values increased only up to ∼1.6 fold of initial value by 9L peaking at 12L and decreased to ∼1.1 fold of the initial value during the dark period (Fig. 1B).

**Fig. 2:**
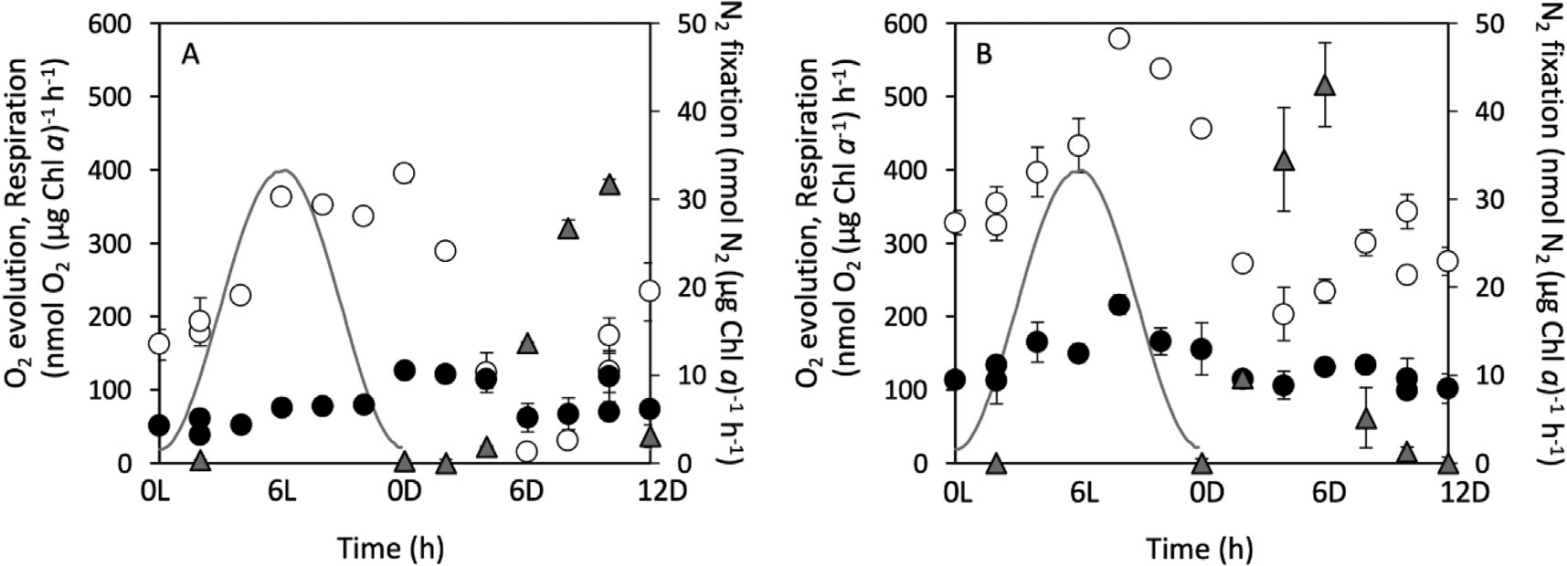
Diel changes of maximal O_2_ evolution rates (open circles), respiration rates (closed circles) and N_2_ fixation rates (grey triangles) in *Crocosphaera* (A) and in *Cyanothece* (B). Grey line illustrates the relative light intensity. Values are averages with error bar showing standard deviations (n = 3).

In both species, the maximal gross O_2_ evolution capacities already started to increase in the middle of the dark phase and reached maxima of 363 nmol O_2_ (µg Chl *a*)^−1^ h^−1^ at L6 and 580 nmol O_2_ (µg Chl *a*)^−1^ h^−1^ at L8 in *Crocosphaera* and *Cyanothece*, respectively (Fig. 2). In both species, O_2_ evolution declined during the early dark phase, but started to increase again in the second half of this period. *Cyanothece* respired actively both in the dark and light phases and its total diurnal respiration was about 2.1-fold higher (1.1 µmol O_2_ (µg Chl *a*)^−1^ light phase^−1^) than that of *Crocosphara* (0.5 µmol O_2_ (µg Chl *a*)^−1^ light phase^−1^) and was 1.2-fold higher overall (2.0 µmol O_2_ (µg Chl *a*)^−1^ day^−1^ in *Cyanothece* compared to 1.7 µmol O_2_ (µg Chl *a*)^−1^ day^−1^ in *Crocosphaera*) (Table 1). *Crocosphaera* started N_2_ fixation gradually so that a marked increase was not measurable until 6 h into the dark (6D) and peaked at 10D with 31.7 ± 0.5 nmol N_2_ (µg Chl *a*)^−1^ h^−1^ (Fig. 2A). On the other hand, *Cyanothece* started to fix N_2_ from the beginning of the dark phase and peaked at 6D (43.0 ± 4.8 nmol N_2_ (µg Chl *a*)^−1^ h^−1^) (Fig. 2B). Over the course of an entire day, *Crocosphaera* fixed 77.1 nmol N_2_ (µg Chl *a*)^−1^, which corresponds to 82 % of the amount fixed by *Cyanothece* (93.7 nmol N_2_ (µg Chl *a*)^−1^).

**Fig. 3:**
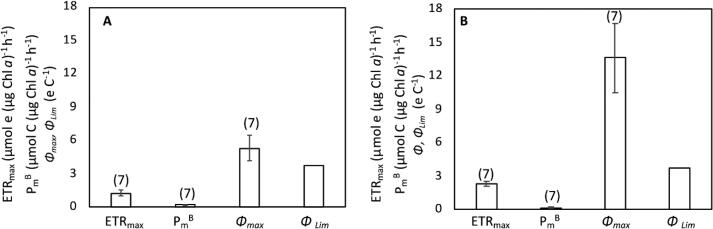
The maximum electron transport rates, ETR_max_, the maximum carbon incorporation rates, P_m_^B^, and the electron demand for carbon fixation under saturating irradiance (*Φ_max_*), and under light limitation (*Φ_Lim_*) in *Crocosphaera* (A) and *Cyanothece* (B). Data are averaged from data points collected during the durnal measurements (i.e. at 2L, 4L, 6L, 8L, 10L, and 12L). Error bar shows standard deviation, the numbers of replicates are shown in parentheses.

The average chlorophyll-normalized maximal carbon incorporation rates P_m_^B^ during the light period were 0.237 ± 0.037 µmol C (µg Chl *a*)^−1^ h^−1^ and 0.175 ± 0.036 µmol C (µg Chl *a*)^−1^ h^−1^ for *Crocosphaera* and *Cyanothece*, respectively, while the corresponding mean PSII-mediated ETR_max_ were 1.260 ± 0.245 µmol e^–^ (µg Chl *a*)^−1^ h^−1^ and 2.290 ± 0.199 µmol e^–^ (µg Chl *a*)^−1^ h^−1^, respectively (Fig. 3). Thus, the electron demand for carbon incorporation (*Φ_max_* = ETR_max_ / P_m_^B^) calculated from these values was 2.5 times higher in *Cyanothece* (13.6 ± 3.1 e C^−1^) compared to *Crocosphaera* (5.3 ± 1.2 e C^−1^), suggesting that the coupling between photosynthetically generated electrons and C fixation at saturating irradiances in *Crocosphaera* is much tighter and more effective compared to *Cyanothece* (Fig. 3). However, the electron requirement for carbon incorporation under non-saturating light intensities (*Φ_Lim_*) were comparable for both *Crocosphaera* (3.7 ± 1.3 e C^−1^) and *Cyanothece* (3.7 ± 0.9 e C^−1^).

The optical densities recorded by the photobioreactor revealed that population dynamics are highly reproducible during consecutive diel cycles (Fig. 1). OD_735_ is a measure of light scattering due to particulate material. It increases when either cell density increases and/or increases in sizes increase, or cells accumulates intracellular storage products such as granules. The non-linear relationships between OD_735_ and cell abundance have been shown previously, both in *Crocosphaera* and *Cyanothece* [26], [34]. The daily changes in cell size as the cells undergo division are also small (<10 %) in both species. Therefore, the diurnal increase in OD_735_ reflects mostly biomass production, specifically the increase in cellular C content as a result of photosynthesis, whereas the nocturnal decrease in OD_735_ is caused by the consumption of cellular C as a consequence of respiration [26], [34]. Since ODs can be influenced by various factors, absolute OD_735_ of two different species may not reflect the same C content. Therefore, to eliminate these differences, we normalized OD_735_ measurements to the corresponding values recorded at 1L. We also recognize that there are other intracellular storage compounds such as cyanophycin, a major nitrogen storage compound, and/or phosphorus granules. However, their content is generally much smaller than that of storage carbohydrates [19], [20], [31] and can be neglected. Under exponential growth conditions, we observed much more dynamic diel changes in OD_735_ in *Crocosphaera* (∼3 μm) compared to *Cyanothece* (∼3 μm). High variability in OD_735_ in *Crocosphaera* may reflect the larger cellular size and/or more peripheral allocation of carbohydrate within these cells [19]. Therefore, we define the rate of changes in OD_735_ as a proxy of C incorporation and consumption; photosynthesis and respiration.

**Fig. 4:**
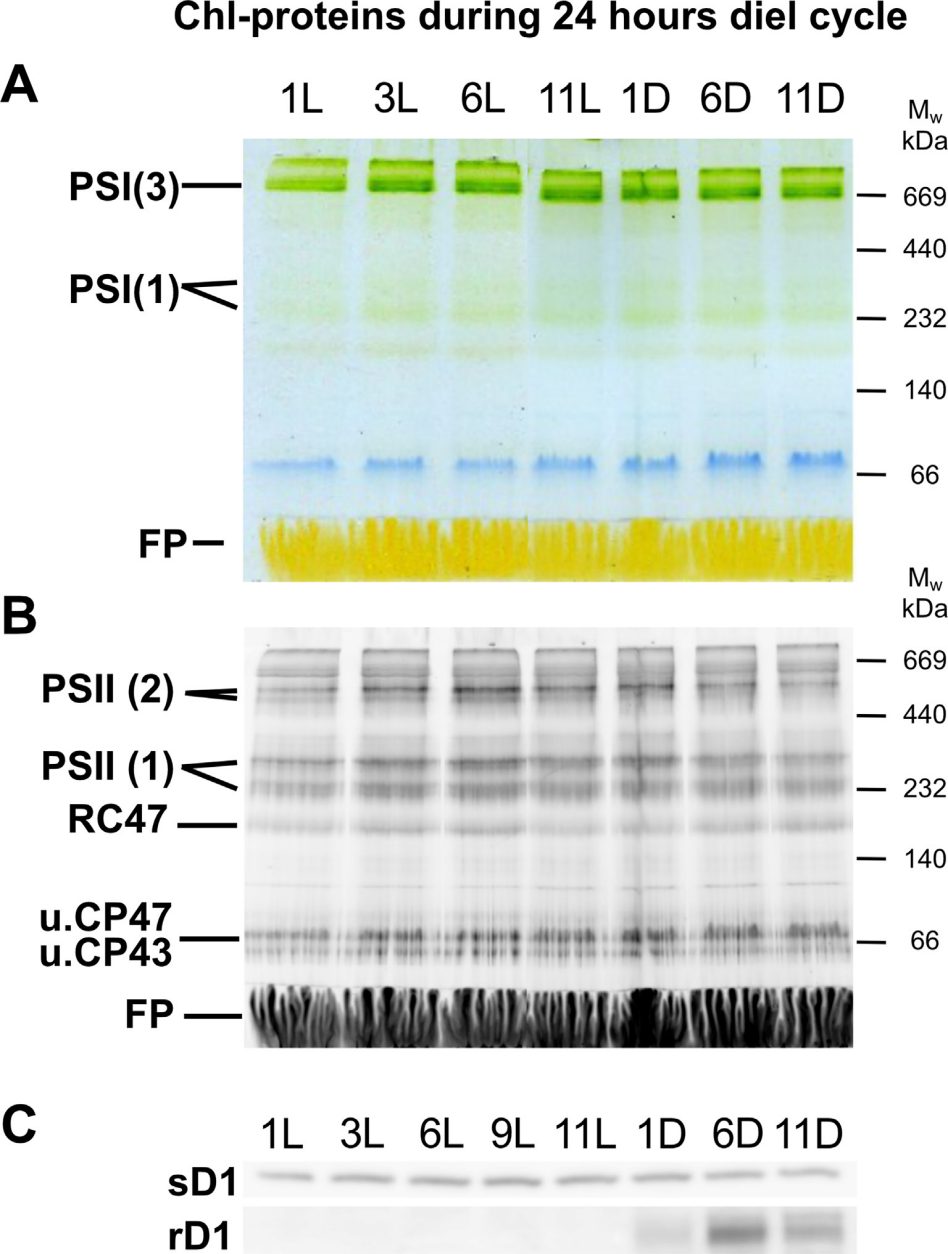
Diel pattern of the abundance of membrane protein complexes and D1 proteins in *Cyanothece* at 1L, 3L, 6L, (9L), 11L, 1D, 6D, and 11D. (A) Isolated membrane proteins were analyzed by CN PAGE; the gel was photographed and (B) scanned to visualize Chl *a* fluorescence with LAS 4000 camera system. Designation of the complexes: PSI (3) and PSI (1), trimeric and monomeric PSI complexes, respectively; PSII (2) and PSII (1), dimeric and monomeric core PSII complexes, respectively; RC47, PSII complex lacking CP43, u.CP43 and u.CP47, unassembled CP43 and CP47; F.P., free pigments. 5 µg of Chl were loaded for each sample. (C) Membranes were analyzed by denaturing SDS-PAGE, gel was electroblotted to PVDF membrane and the membrane was probed with antibodies specific for the standard D1 (sD1) and rD1. 2 µg of Chl were loaded for each sample.

Our experiments were designed to compare the photosynthetic and N_2_ fixation capacity of the two studied strains under optimal growth conditions, i.e. during exponential growth, without any nutrient or light limitation. Under such conditions, the diel pattern in photosynthetic activities and N_2_ fixation in *Crocosphaera* and *Cyanothece* showed similarities, but also pronounced differences. The latter of which were as follows: firstly, *Crocosphaera* maintained high O_2_ evolving capacity even at the very end of the light phase, whereas *Cyanothece* reduced O_2_ evolving capacity in parallel with the decreasing light intensity after 8L (Fig. 2). Secondly, *Crocosphaera* lost the capacity for photosynthetic O_2_ evolution when actively fixed N_2_, whereas *Cyanothece* still retained its capacity to evolve O_2_ in the middle of the dark phase (Fig. 2). This uncoupling of the photosynthetic capacity from N_2_ fixation in the dark in *Crocosphaera* is well documented [26], [33] and can be explained by inactivation of PSII complexes, their monomerization and disassembly, most probably related to the decreased protein synthesis [26]. The nocturnal decline of PSII activity has also been reported for *Cyanothece* [38]. However, analysis of the membrane protein complexes using CN PAGE (Fig. 4) revealed that the monomerization and disassembly was not as significant in that microorganism as what was observed for *Crocosphaera* [26]. As observed earlier [38], non-functional rD1 protein was accumulated specifically during dark phase, similar to that observed in *Crocosphaera* [26].

Finally, the timing of N_2_ fixation also differed in the two cyanobacteria. *Cyanothece* started to fix N_2_ shortly after the start of the dark period, whereas *Crocosphaera* started to fix N_2_ only after 4 h of darkness. We assume that the swift shift from photosynthesis to N_2_ fixation in *Cyanothece* is supported by the observed relatively high respiration rates, which was comparable in the dark and light phases (Fig. 2B, Table 1). In contrast, the respiration rates in *Crocosphaera* during the light phase was less than half of its dark rates (Table 1). Besides, the rate of respiration compared to gross O_2_ evolution during the light phase was higher in *Cyanothece* (36 %), compared to that of *Crocosphaera* (25 %). These results suggest that a balance between C incorporation and consumption may be the main reason of the observed smaller diel OD dynamics in *Cyanothece* (Fig. 1). Interestingly, the duration of active N_2_ fixation was about 6 h in both species, and active N_2_ fixation stopped about 2 h before the start of the daylight phase.

The lower ETR_max_ coupled with higher P_m_^B^ in *Crocosphaera* suggests that this species is more efficient in C incorporation under saturated irradiance compared to *Cyanothece*, as shown by low *Φ_max_*. However, under light-limiting conditions, the electron requirement was identical for both strains (Fig. 3), which suggests that *Crocosphaera* that contains less thylakoid membranes as well as chlorophyll per cell captures electrons using larger light-harvesting antenna compared to *Cyanothece*. On the other hand, assuming that 8 electrons are necessary to fix one N_2_ [13] in both *Crocosphaera* and *Cyanothece,* ETR at given irradiance suggested that 9.1 %, and 9.3 % of the transported electrons are devoted to N_2_ fixation, respectively. Thus, the efficiency of electron utilization in nitrogen fixation is comparable in these species.

## 4 Simulated competition between *Crocosphaera* and *Cyanothece* in a simple ecosystem model

**Fig. 5:**
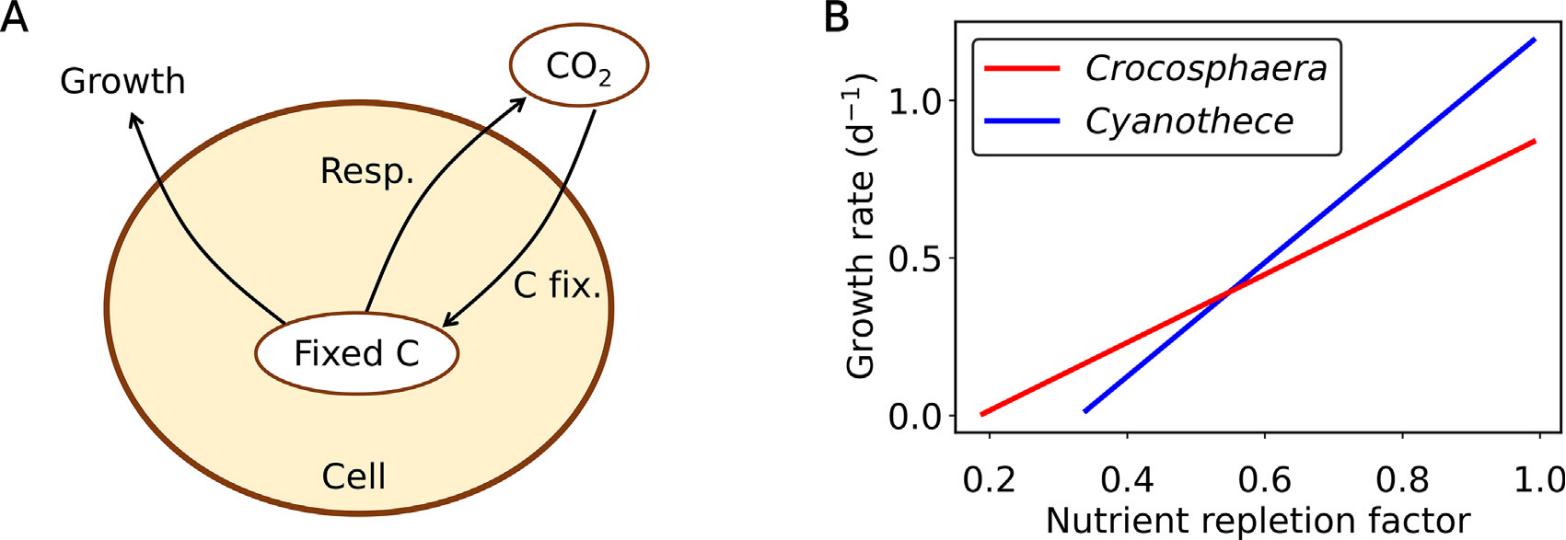
A simplified model to describe the dependence of microbial growth of *Crocosphaera* and *Cyanothece* on the nutrient repletion factor. (A) A schematic representation of a cyanobacterial cell, showing the major C routes according to the model CFM-CC (for details, see the text). Resp., respiration; C fix., C fixation. In this model, the balance of C fixation and respiration determines the cellular growth. (B) Results of the model calculations. The nutrient repletion factor affects the growth of *Crocosphaera* and *Cyanothece differently*.

The above described results showed that *Cyanothece* exhibits higher photosynthesis as well as dark respiration rates when compared with *Crocosphaera*. The question arises whether these differences in C-related metabolisms could explain their ecological success in different niches. To address this question, we developed a simple metabolic model for phytoplankton (CFM-CC (Fig. 5A), see also Materials and Methods). The model is based on a simple equation but has an aspect of a coarse-grained model [18], resolving key metabolic pathways including C fixation, respiration and growth. The residual C after C fixation and respiration is converted to biomass of new cells *via* cell division (growth). In general, in an interspecies competition, when two species use the same resources, the faster growing strain outcompetes the other one.

**Fig. 6:**
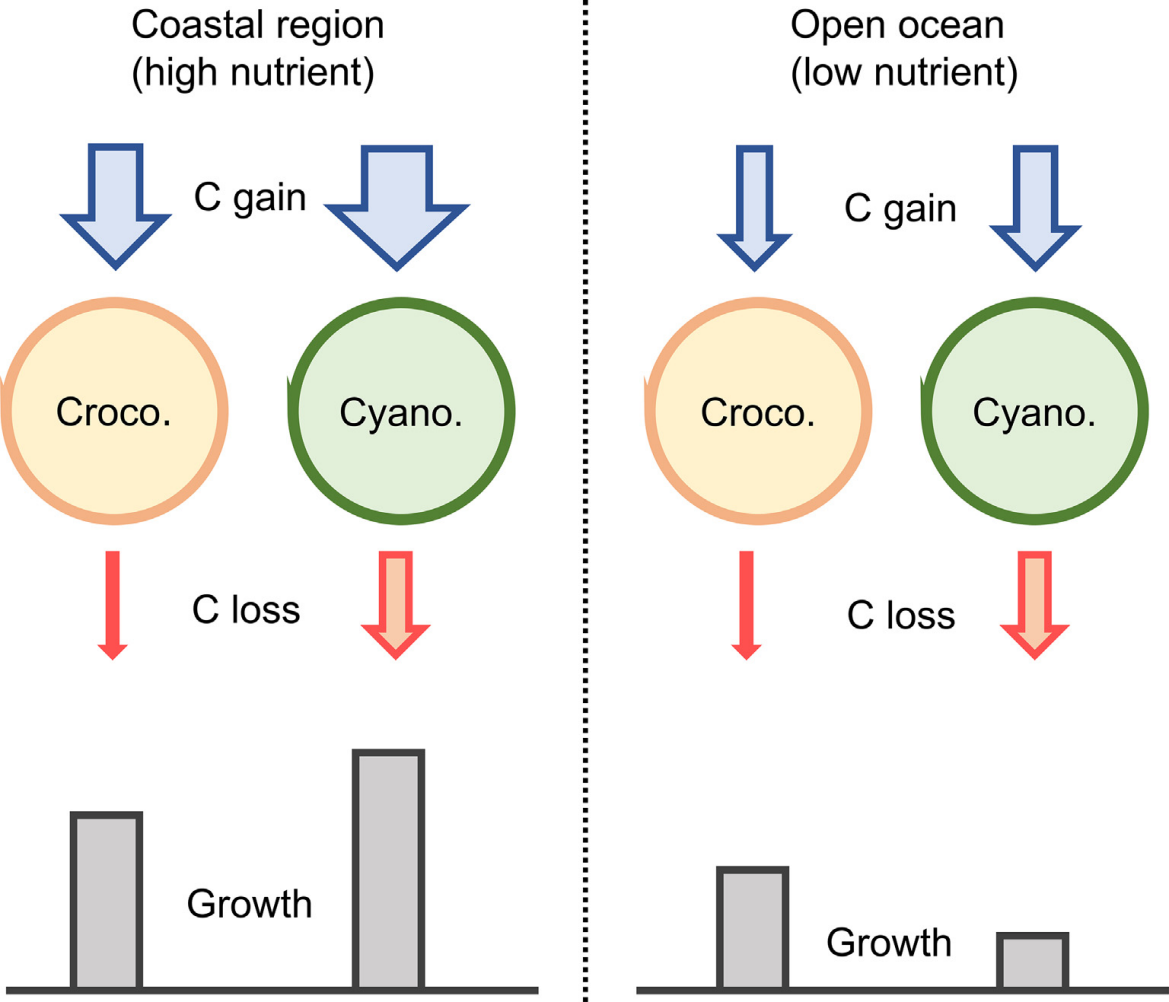
Schematic model interpretations and implications. Growth is depicted as a sum of C gain and C loss. In coastal regions where nutrient concentrations are generally high, *Cyanothece* (Cyano.) has higher growth rates due to its high C fixing capability. However, in open ocean *Crocosphaera* (Croco.) has an advantange because of it low C loss.

We calculated growth rates of *Crocosphaera* and *Cyanothece* based on the CFM-CC model and plotted as a function of nutrient repletion factor in Fig. 5B. Importantly, this factor indicates how nutrient limitation influences the rate of C fixation. The higher this factor the smaller the nutrient limitation. For example, at nutrient repletion factor of 0.3 and 0.7, 30 % and 70 % of maximum photosynthesis occurs due to relatively high and low nutrient limitations, respectively. Under nutrient-replete conditions as are frequently found in its coastal environment (characterized with high nutrient repletion factor), *Cyanothece* has higher growth rates indicating an ecological advantage under such conditions, which allow the organism to take advantage of their inherently high rate of photosynthesis and an overall higher maximum photosynthesis rate (Table 1). In contrast, under nutrient limiting conditions, *Crocosphaera* predominates as indicated by higher growth rates under low nutrient repletion factors therefore enabling it to occupy the niche of low-nutrient open ocean waters due to its low respiration rates coupled with a highly efficient metabolism. These conclusions are summarized in Fig. 6. Despite the simple parameterizations, our results clearly show a niche separation of these microorganisms, underpinning the significance of differences in C metabolisms in shaping of ecological niches.

In this study, we focused on the growth rate, but factors affecting the mortality rate may affect the organismal competition. For example, if the grazing patterns for these organisms are different, it may affect the competition. However, such differentiated grazing for these organisms have not been reported under the same condition. If the size of these organisms is the same, under the same environmental conditions, grazing rates for these organisms are likely to be similar. Given the identical grazing/mortality rates, the competition is mainly governed by the growth rate. At the same time, uncertainties in grazing rates are large; the grazing rates vary from nearly zero to as high as 0.7 d^−1^ for *Crocosphaera* [10], [43], whereas grazing rates for *Cyanothece* seem to be slightly more stable (0.18–0.58 d^−1^) [9]. Pinning down the effect of grazing will ultimately require additional experiments for these two organisms and their potential grazers under a set of identical growth conditions.

## 5 Conclusions

Overall, our integrated study of laboratory scale measurements showed that highly reproducible diel changes in OD_735_ is a proxy for population metabolic dynamics. The observed diel changes were much more dynamic in *Crocosphaera* compared to *Cyanothece*. This dynamic change is possibly a consequence of the strict temporal segregation of photosynthesis and respiration in *Crocosphaera.* A simple ecosystem model with two competing species suggested that differences in C incorporation and consumption may lead to different niche acquisition: High C fixing capability enables *Cyanothece* to grow actively in coastal waters, and low C loss enables *Crocosphaera* to survive in oligotrophic water.

## Declaration of Competing Interest

The authors declare that they have no known competing financial interests or personal relationships that could have appeared to influence the work reported in this paper.
