# Supplementary material for: The balance between photosynthesis and respiration explains the niche differentiation between Crocosphaera and Cyanothece
Source: Comput Struct Biotechnol J. 2022 Nov 17;21:58–65. doi: 10.1016/j.csbj.2022.11.029 (PMC9732122; doi:10.1016/j.csbj.2022.11.029)
Supplement: Supplementary data 1 [file mmc1.docx]

| Table S1. Variables, units and definitions for Equation 2 in the main text | | |
| --- | --- | --- |
| Variables | Unit | Definition |
| $\mu_{i}$ | d^-1^ | Growth rate of microorganism *i* |
| *i* | - | Represent different phytoplankton, *Cro* = *Crocosphaera*, *Cya* = *Cyanothece* |
| $P_{Max}^{i}$ | d^-1^ | Maximum C fixation rate for microorganism *i* |
| $m_{i}$ | d^-1^ | Maintenance of microorganism *i* |
| $f_{N}$ | Dimensionless unit | Nutrient repletion factor, ranging from 0 (deplete environment) to 1 (replete environment) |

| Table S2. Variables, units and values for Equation 2 in the main text | | |
| --- | --- | --- |
| Variables | Unit | Value |
| $P_{Max}^{Cro}$ | d^-1^ | 1.08 |
| $P_{Max}^{Cya}$ | d^-1^ | 1.81 |
| $m_{Cro}$ | d^-1^ | 0.2 |
| $m_{Cya}$ | d^-1^ | 0.6 |
